# The More Similar, the Healthier: The Effect of Perceived Parent-Child Facial Resemblance on Parental Physical Health

**DOI:** 10.3389/fpsyg.2018.02739

**Published:** 2019-01-11

**Authors:** Quanlei Yu, Yafei Guo, Lin Zhang, Jianwen Chen, Xiaopeng Du, Xinhui Wei, Zhijin Zhou, Shumin Liu, Xinlei Gao

**Affiliations:** ^1^Key Laboratory of Adolescent Cyberpsychology and Behavior of the Ministry of Education and School of Psychology, Central China Normal University, Wuhan, China; ^2^ School of Entrepreneurship and Management, ShanghaiTech University, Shanghai, China; ^3^Institute of Health, Shandong University of Traditional Chinese Medicine, Jinan, China; ^4^Graduate School of Education, Huazhong University of Science and Technology, Wuhan, China; ^5^School of Psychology, Beijing Normal University, Beijing, China; ^6^Institute of Psychology, Chinese Academy of Sciences, Beijing, China; ^7^Beijing Changping District Huilongguan Central Primary School, Beijing, China

**Keywords:** perceived parent-child facial resemblance, paternal uncertainty, physical health, trait anxiety, parent gender

## Abstract

Parent-child facial resemblance (PCFR) is one of the direct cues used to assess the genetic relationship between two individuals. Due to the inner fertilization of humans, fathers are liable to suffer from paternal uncertainty. When a father perceives low father-child facial resemblance, he would become anxious, which is detrimental to his immune system and physical health. For a mother, however, she can assure her genetic relationship to her children and does not need any external cues to verify her maternity. Thus, the mother-child facial resemblance does not influence the mothers’ physical health. To test these hypotheses, we examined the moderating effect of parental gender and the mediating effect of trait anxiety on the relationship between PCFR and physical health of parents. The results showed that fathers’ PCFR positively predicted their physical health, whereas the mothers’ PCFR failed to show any predicting effect on mothers’ physical health. Furthermore, trait anxiety mediated the relationship between fathers’ PCFR and their physical health. The implications for paternal uncertainty, gender difference, and public policy were discussed.

## Introduction

### Paternal Uncertainty

Darwin (1859) established that all species of life have evolved with two fundamental motives, survival and reproduction. Decades later, modern evolutionary psychologists proposed further that reproduction is the objective of survival (Kenrick et al., 2010). Due to humans’ internal fertilization, women are confident in their genetic connection to offspring (Gaulin and Schlegel, 1980). Men, however, have always been less sure whether they are genetically related to their offspring since adultery is rife in early human society. Thus, men, rather than women, may face the risk of paternal uncertainty (Larsen and Buss, 2008; D. Buss, 2014). To cope with this uncertainty, men tend to seek a variety of cues that indicate their genetic connection to offspring, such as the perceived spouse’s fidelity (Flinn, 1988; Apicella and Marlowe, 2004), facial resemblance, and body odor resemblance (Alvergne et al., 2009, 2010; Bressan et al., 2009). Facial resemblance is one of the direct cues that has been frequently documented in literature (Burch and Gallup, 2000; Apicella and Marlowe, 2004; DeBruine, 2005; Alvergne et al., 2007, 2009, 2010; Krupp et al., 2008; Lewis, 2011; Yu et al., 2016, 2017; Billingsley et al., 2018). Past research has established three ways to access facial resemblance, namely self-rating (Burch and Gallup, 2000; Apicella and Marlowe, 2004; Alvergne et al., 2010; Lewis, 2011; Yu et al., 2016, 2017; Billingsley et al., 2018), third-party rating (Alvergne et al., 2007, 2009; Lewis, 2011), and the morphological method (DeBruine, 2005; Krupp et al., 2008). In terms of ecological validity, self-rating is more favorable compared to the other two methods (Yu et al., 2016). Thus, the present study adopted the self-report method to access parent-child facial resemblance.

### Psychological Consequences of Paternal Uncertainty

To ensure genetic transmission across generations, humans tend to exhibit higher kin altruism than non-kin altruism (Hamilton, 1964; Anderson, 2005; Tifferet et al., 2016; Bernhold and Giles, 2017). Parents invest a large amount of material and emotional resources into their offspring until their children grow up and are able to live by themselves. However, the risk of paternal uncertainty might discourage fathers’ familial investment. Past studies have shown that the higher facial similarity that fathers perceived with their children, the more resources they would invest in their offspring (Alvergne et al., 2009; Yu et al., 2017). Some research has also suggested that high facial similarity was associated with a low rate of domestic violence (Burch and Gallup, 2000; Platek et al., 2002; Heijkoop et al., 2009).

Notably, to reduce paternal uncertainty, males value sexual fidelity highly in the mating process. Scholars have demonstrated that males would show a great deal of upset and anxiety when imagining their partners’ sexual infidelity (e.g., having passionate sexual intercourse with other males) (D. M. Buss et al., 1992). Once adultery has been confirmed, males would hardly forgive their partners and are more prone to terminate their current relationship (Shackelford et al., 2002). Likewise, if there is a low father-child facial resemblance, males might be uncertain about their partners’ fidelity and get anxious and upset as well. Besides, taking care of children is persistent and lasting. Fathers might have to live with this uncertainty for years unless they have the courage to verify their genetic connection. Thus, Yu et al. (2016) deemed that the lasting stress and nervousness, induced by the low father-child facial resemblance, would contribute to father’s trait anxiety, which is defined as “an emotional state that included feelings of apprehension, tension, nervousness, and worry accompanied by physiological arousal” (Spielberger, 1983). According to the pathogenesis of psychosomatic diseases, the enduring of trait anxiety could be harmful to people’s physical health (Durand and Barlow, 2012). Thus, we argued that the perceived facial resemblance would not only influence fathers’ trait anxiety but also affect their physical health through the mediating path of trait anxiety.

Specifically, fathers who perceive low levels of father-child facial resemblance would show low function in their immune system after suffering from the long-time trait anxiety. Previous studies have shown that when individuals perceive stress or threats, the Hypothalamic–Pituitary–Adrenocortical (HPA) axis is activated as the response center (Durand and Barlow, 2012). Specifically, once a stressor or threat is detected, the hypothalamus excretes the adrenal hormone releasing factor (CRF), stimulating the release of the adrenal cortisol hormone, hormone cortisol, and other hormones from the adrenal gland (Kring et al., 2013). Thus, humans could cope with external pressure and threats effectively by adjusting hormonal release according to the stressor (Dickerson and Kemeny, 2004; Jaremka et al., 2013). When the stressors are lasting and uncontrollable, there are greater hormonal changes and a longer recovery duration in the release of cortisol and adrenocorticotropin (Henry and Grim, 1990; Sapolsky, 1993; Dickerson and Kemeny, 2004). Additionally, past research manifested that chronic anxiety might suppress an individual’ s immune system (Koh and Lee, 1998) and physical activity (Stubbs et al., 2017). Anxious people would display dysregulation in the 5-HT system (Chang et al., 2003), attenuation in norepinephrine (Geracioti et al., 2001), an increase in cortisol concentration (Dickerson and Kemeny, 2004), and acceleration of catecholamine (Levy et al., 1985). All these physiological reactions are detrimental to the human immune system (Koh and Lee, 1998; Dickerson and Kemeny, 2004), and thereby, their physical health (Durand and Barlow, 2012), resulting in poor appetite, insomnia, and muscle pain (Smoller et al., 2007; Roy-Byrne et al., 2008).

**FIGURE 1:**
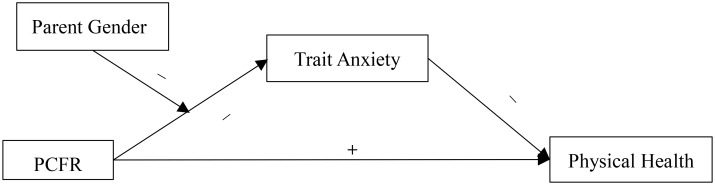
The effect of perceived parent-child facial resemblance (PCFR) on parental physical health: a moderated mediation model.

On the basis of the above research, we hypothesized that trait anxiety would mediate the effect of perceived parent-child facial resemblance (PCFR) on physical health. Meanwhile, given that mothers are confident in their maternity, they would not suffer from trait anxiety, and their physical health would be unaffected by perceived PCFR. That is, parental gender would be a boundary condition for the mediation model (see Figure 1).

### The Present Research

The present work recruited parents of primary school students to explore the mediating effect of trait anxiety on the relationship between perceived parent-child facial resemblance and physical health. Providing that parent age (Nakazato and Shimonaka, 1989) and family socioeconomic status (SES) (Pinquart and Sorensen, 2000; Johnson and Krueger, 2006) might affect parental physical health, we controlled parent age and family SES in the two studies. Additionally, since it is difficult to figure out a plausible composite score of parent-child facial resemblance in non-single-child families (Yu et al., 2016), we excluded data of parents from non-single -child families.

## Materials and Methods

### Participants

This study was reviewed and approved by Beijing Normal University Ethics Committee, all methods were performed in accordance with government regulations and laboratory’s policies, and all respondents signed informed consent. One hundred and ten parents from 110 independent single-child families were recruited from a primary school in Northern China. Of these participants, 10 were excluded, including four single-parent-family parents and six remarried-family parents. Finally, 100 cases (54 fathers and 46 mothers; *M*_age_ = 38.98, *SD*_age_ = 3.30) remained in the current analysis.

### Materials

#### Facial Resemblance Measure

Participants completed the two items of Perceived Parent-Child Facial Resemblance scale (Yu et al., 2016). The scale contains two items: “In your opinion, how much does the youngest child look like you?” and “In your friends’ or family members’ views, how much does the youngest child look like you?”. The scale was rated on a 10-point Likert scale, ranging from 1 (not at all) to 10 (completely). The correlation between the two scale items was 0.83 in this study.

#### Physical Health Measure

Participants’ physical health measure was derived from the subscale of “physical symptom and organ function” in the Self-Rated Health Measurement Scale (Wang et al., 1999; Xu et al., 2000). This subscale contains seven items, for example, “How about your appetite?”, “How about your sleep?”, “Do you experience some extended pain?”. Participants rated on an 11-point Likert scale, ranging from 0 (*very poor*) to 10 (*very well*). The scale had high reliability and validity (Xu et al., 2000). The Cronbach’s α was 0.85 and the split-half reliability was 0.86 in this study.

#### Trait Anxiety Measure

We adopted the Trait Anxiety subscale in the State-Trait Anxiety Inventory (STAI) (Spielberger, 1983) to measure participants’ trait anxiety. The subscale contains a total of 20 items (e.g., “I feel safe,” “I have a lack of confidence,” and “I am a calm person”), and participants need to rate on a 4-point Likert scale, ranging from 1 (*not at all*) to 4 (*very much*). The subscale has been demonstrated to have satisfactory reliability and validity (Spielberger, 1983). The Cronbach’s α was 0.90 and the split-half reliability was 0.92 in this study.

#### Demographic Measures

Participants’ demographic information was gathered, including gender, age, the years of education of participants and their spouse, annual household income, and the number of children.

### Procedures

Firstly, we recruited six teachers and trained them as research assistants. Secondly, research assistants delivered the questionnaires to primary students in class and instructed them to take home to their fathers or mothers. Finally, the primary students brought back the completed questionnaires to the research assistants.

### Data Analysis

Firstly, the macro PROCESS model 1 was used to examine the moderating effect of parent gender on the relationship between perceived parent-child facial resemblance and parental physical health. The macro PROCESS model 7 was used to explore the moderating mediation effect on the relationship between perceived parent-child facial resemblance and parental physical health. To ensure the integrity of the cases, this study adopted the listwise method to deal with missing values.

Harman’s single factor test was used to test the common method bias. The results showed that the first factor explained by the unrotated and rotated variables was 27.42 and 17.06%, respectively. They were both below the critical 40% threshold. Therefore, the common method bias effect was not significant in this study.

## Results

### Preliminary and Descriptive Analysis

As suggested by Kraus et al. (2009), we standardized and averaged participants’ education, their spouses’ education and annual household income as participants’ family SES. To facilitate the subsequent regression analysis, the gender of parents and children was dummy coded (female = 1, male = 0).

As shown in Table 1, trait anxiety correlated negatively with physical health. However, neither trait anxiety nor physical health was significantly associated with perceived PCFR. One possible explanation is that the correlation is too small among mothers since they are sure of their genetic contribution to offspring and do not need extra cues, such as facial resemblance, to verify their maternity. Thus, we failed to detect any significant correlation between perceived PCFR and other variables in the whole sample. In addition, the results from sub-groups supported our aforementioned notion. The correlations in fathers’ sub-group was significant and the coefficient was moderate in magnitude (*r*_perceivedPCFR-traitanxiety_ = -0.43, *p* < 0.001; *r*_perceivedPCFR-traitanxiety_ = 0.38, *p* = 0.004). And the correlation in mothers’ sub-group was weak and not significant (*r*_perceivedPCFR-traitanxiety_ = 0.002, *p* = 0.99; *r*_perceivedPCFR-traitanxiety_ = -0.12, *p* = 0.43).

**Table 1:** Descriptive statistics and correlations among variables in study 2 (*N* = 100).

	1	2	3	4	5
1. Age	–				
2. Family SES	0.01	–			
3. Trait anxiety	0.07	–0.19^†^	–		
4. Physical health	–0.06	–0.24**	–0.44***	–	
5. Perceived PCFR	0.004	0.05	–0.20^†^	0.16	–
*M*	38.98	0.00	1.94	6.79	7.18
*SD*	3.30	2.34	0.40	1.47	1.68


*^∗∗∗^*p* < 0.001; ^∗∗^*p* < 0.01; and ^†^*p* < 0.1.*

### The Moderating Effect of Parent Gender

To examine the moderating effect of parent gender on the relationship between perceived PCFR and physical health, we adopted model 1 in the PROCESS macro (Hayes, 2013). A regression analysis was conducted, with perceived PCFR being the predictor, physical health being outcome variable, parent gender being the moderator, and parent age and family SES being the control variables.

**FIGURE 2:**
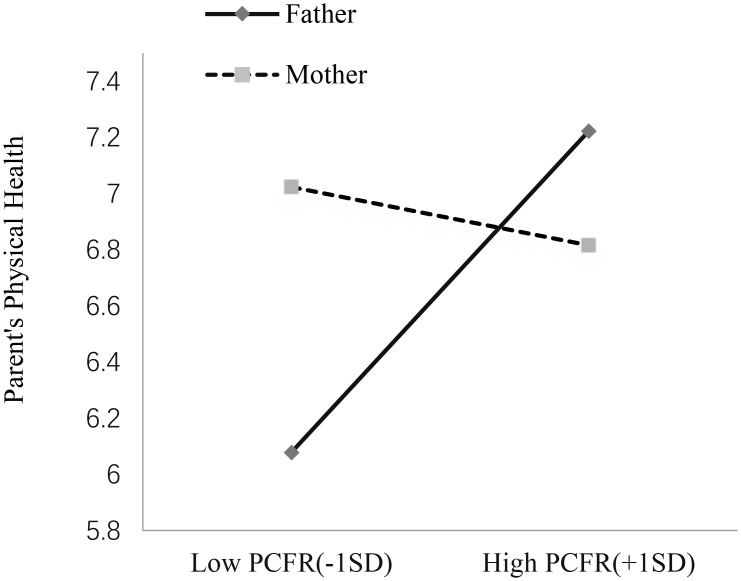
The moderating effect of parent gender on the relationship between PCFR and parental physical health in this study. The figure depicts the physical health score corresponding to –1SD and +1SD of PCFR for both parents. The slope was significant for fathers, but not for mothers.

As shown in Table 2, a significant interaction between parent gender and perceived PCFR emerged. Then, a simple slope effect test was conducted (see Figure 2). In the fathers’ group, perceived father-child facial resemblance significantly predicted fathers’ physical health, *B* = 0.34, *SE*_B_ = 0.12, 95% bootstrap CI = [0.004, 0.11] (not including zero), *t*(94) = 2.95, *p* < 0.01, η^2^ = 0.09. Whereas, in the mothers’ group, the effect of perceived father-child facial resemblance on mothers’ physical health was not significant, *B* = -0.062, *SE*_B_ = 0.123, 95% bootstrap CI = [-0.30, 0.18] (including zero), *t*(94) = -0.50, *p* = 0.61.

**Table 2:** The moderating effect of parent gender on the relationship between perceived PCFR and physical health.

Predictor variables	Outcome variable: physical health (*N* = 100)	
	*B*	*SE_B_*
Intercept	4.55*	2.04
Age	–0.01	0.05
Family SES	–0.15*	0.06
Perceived PCFR	0.34**	0.12
Parent gender	3.15*	1.24
Perceived PCFR × Parent gender	–0.40*	0.17
*ΔR^2^* of interaction	0.05**	


*^∗∗∗^*p* < 0.001, ^∗∗^*p* < 0.01, and ^∗^*p* < 0.05.*

### The Mediated Moderation Analysis

To further examine the moderated mediation model, we used model 7 in the macro PROCESS (Hayes, 2013) to analyze the data, with perceived PCFR being the predictor, parental gender being the moderator, trait anxiety being the mediator, parental physical health being the outcome variable, and participants’ age and family SES being the control variables.

**FIGURE 3:**
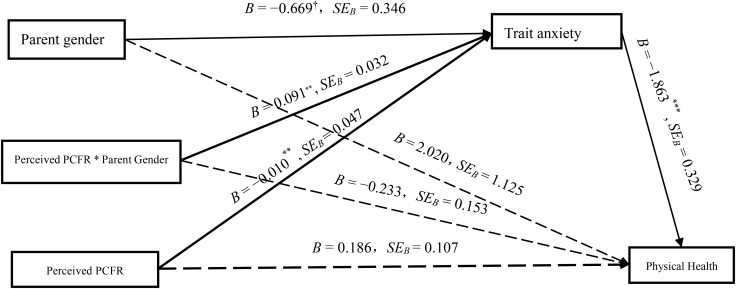
The moderating effect of parent gender and mediating effect of trait anxiety on the relationship between perceived PCFR and parental physical health.

As shown in Figure 3, results showed that parental trait anxiety fully mediated the product of parental gender and perceived PCFR, *B* = -0.18, *SE_B_* = 0.01, 95% bootstrap CI = [-0.38, -0.01] (not including zero). Among fathers, trait anxiety fully mediated the relationship between the perceived father-child facial resemblance and their physical health, *B* = 0.16, *SE_B_* = 0.06, 95% bootstrap CI = [0.07, 0.29] (not including zero). However, the mediating effect of mothers’ trait anxiety was not significant, *B* = -0.02, *SE_B_* = 0.06, 95% bootstrap CI = [-0.15, 0.10] (including zero).

## Discussion

The present study examined a moderated mediation model of the relationship between perceived PCFR and physical health. Results showed that fathers’ perceived PCFR, rather than the mothers’ perceived PCFR, had a positive effect on their physical health, and this effect could be accounted for by trait anxiety. Consistent with the previous study (Yu et al., 2016), our findings supported the paternal uncertainty hypothesis. In sum, PCFR not only regulates parental investment (Alvergne et al., 2009, 2010; Yu et al., 2017) but also impacts parental physical health. Yet PCFR’s negative impact on physical health is contingent on parent gender.

### The Costs and Benefits of Parenthood

While delivering and raising children, mothers tend to do more than fathers in human society. During the 9-month pregnancy, mothers would have to compromise on their work. Most mothers, especially in underdeveloped countries, also bear the responsibility of feeding and taking care of the children. In a word, in terms of the costs of parenthood, fathers seem to sacrifice less than mothers (Trivers, 1972; D. Buss, 2014). From the perspective of benefits, however, mothers tend to reap more than do fathers. On the one hand, due to humans’ inner fertilization, mothers are confident in their genetic contribution to offspring, while fathers are likely to suffer from paternal uncertainty (Gaulin and Schlegel, 1980). That is, mothers’ genes are more likely than fathers’ to be successfully transmitted to the next generation. On the other hand, paternal uncertainty might trigger trait anxiety (Yu et al., 2016), leading to poor physical health. Thus, the parental investment seems to be fair in that mothers invest more and reap more, whereas fathers pay less and gain less.

It is worth noting that the present findings might contribute to the understanding of the gender gap in longevity. Generally, women’s average lifespan is approximately 5 to 6 years longer than males’ longevity (Liu et al., 2003; Meara et al., 2008). Past research indicated that the gender gap in longevity was related to physiological factors, such as aging rate and hormones (Liu et al., 2003). Liu et al. (2003) found that estrogen offered women some immunity from cardiovascular disease, resulting in their slower aging rate and longer longevity. Other researchers showed that sex chromosomes could also provide an account of the gender gap in longevity (Christensen et al., 2000; Sandovici et al., 2004). Apart from these physiological accounts, the present research seems to offer a psychological alternative. We found that men are liable to suffer from lasting anxiety and stress if they could not get rid of the risk of paternal uncertainty, fearing that their children may inherit others’ genes. Given that lasting anxiety and stress does harm to the human immune system and physical health, men’s longevity would be reduced. For women, however, they are assured that their children inherit their genes and do not feel anxious about maternal certainty. Thus, women tend to enjoy longer longevity compared to men. Moreover, due to the risk of paternal uncertainty, fathers with low PCFR may be less involved in child raising than mothers (Alvergne et al., 2009; Yu et al., 2017). Reap as you sow. When people get old, we speculate that children would return more to mothers than fathers. If so, this might to a certain extent further amplify the gender differences in parent health and longevity. But this speculation still awaits rigorous examinations in future research.

Additionally, our research also has some implications for public policy. Abundant research has established that females are disadvantaged in modern society (Inglehart et al., 2008). Non-government organizations, such as the National Organization for Women, and women-associated social activities all endeavor to call for and improve women’s physical and psychological well-being. However, little attention has been paid to men’s health. Our research showed that men, especially those who have low facial similarity perception with their children, are suffering from paternal uncertainty and lasting anxiety. To a certain extent, they are also disadvantaged, and their physical and mental health also warrant social attention.

### Limitations and Future Research

The present findings supported our hypothesis that fathers’ perceived PCFR could predict their physical health and trait anxiety was the underlying mechanism. However, there are still some limitations. First, the present findings were derived from static data, which was deficient in demonstrating casual relationships. Thus, further studies could adopt a longitudinal method or experiment paradigm to examine the causality between PCFR and physical health. Second, the present study theorized that trait anxiety impacted fathers’ physical health through the undermining of immune system function. However, we failed to directly measure the level of the immune system and test this idea in the present study. Further studies might pay more attention to the effect of perceived PCFR on the immune system function. Third, this study used a self-rating questionnaire to assess participants’ physical health. Further research should include some objective indexes (i.e., healthcare expenditures, Health records) to replicate and extend the present findings (Mikolajczak and Van Bellegem, 2017). Finally, parent-child facial resemblance is one of the many cues that could indicate fathers’ paternal uncertainty. Yet it is not the necessary and only condition. Paternity can be verified by many other cues (e.g., spouse’s fidelity, the resemblance of body odor). Accordingly, the future study might examine the effect of multiple cues while interpreting the relationship between paternal uncertainty and parental health.

## Conclusion

On the basis of the hypothesis of paternal uncertainty, this study examined the moderating effect of parental gender and the mediating effect of trait anxiety on the relationship between perceived PCFR and physical health. Results showed that fathers,’ but not mothers,’ perceived PCFR positively affected their physical health, and this effect was accounted for by trait anxiety. These findings supported the hypothesis of paternal uncertainty and offered new insight into the gender differences in parental health.

## Author Contributions

QY, YG, LZ, XW, XD, and ZZ designed this study and drafted the manuscript. QY, LZ, XW, SL, and XG performed the research. QY, YG, and LZ analyzed the data. All authors approved the final version of manuscript for submission.

## Conflict of Interest Statement

The authors declare that the research was conducted in the absence of any commercial or financial relationships that could be construed as a potential conflict of interest.
